# Household perceptions towards a redistributive policy across health insurance funds in Tanzania

**DOI:** 10.1186/s12913-015-0761-z

**Published:** 2015-03-15

**Authors:** Eunice N Chomi, Phares GM Mujinja, Kristian Hansen, Angwara D Kiwara, Ulrika Enemark

**Affiliations:** Muhimbili University of Health and Allied Sciences, Dar-es-Salaam, Tanzania; Aarhus University, Aarhus, Denmark; London School of Hygiene and Tropical Medicine, London, UK

**Keywords:** Health insurance, Redistributive mechanisms, Solidarity

## Abstract

**Background:**

The Tanzanian health insurance system comprises multiple health insurance funds targeting different population groups but which operate in parallel, with no mechanisms for redistribution across the funds. Establishing such redistributive mechanisms requires public support, which is grounded on the level of solidarity within the country. The aim of this paper is to analyse the perceptions of CHF, NHIF and non-member households towards cross-subsidisation of the poor as an indication of the level of solidarity and acceptance of redistributive mechanisms.

**Methods:**

This study analyses data collected from a survey of 695 households relating to perceptions of household heads towards cross-subsidisation of the poor to enable them to access health services. Kruskal-Wallis test is used to compare perceptions by membership status. Generalized ordinal logistic regression models are used to identify factors associated with support for cross-subsidisation of the poor.

**Results:**

Compared to CHF and NHIF households, non-member households expressed the highest support for subsidised CHF membership for the poor. The odds of expressing support for subsidised CHF membership are higher for NHIF households and non-member households, households that are wealthier, whose household heads have lower education levels, and have sick members. The majority of households support a partial rather than fully subsidised CHF membership for the poor and there were no significant differences by membership status. The odds of expressing willingness to contribute towards subsidised CHF membership are higher for households that are wealthier, with young household heads and have confidence in scheme management.

**Conclusion:**

The majority may support a redistributive policy, but there are indications that this support and willingness to contribute to its achievement are influenced by the perceived benefits, amount of subsidy considered, and trust in scheme management. These present important issues for consideration when designing redistributive policies.

## Background

The Tanzanian health care system is financed from various sources, including taxation, donor funding, out-of-pocket (OOP) payments, and prepayment schemes. During the 2011/12 financial year, government funding was the dominant source of financing for the health sector, contributing 59% to health expenditures. During the same period, donor funding contributed 41% to the health sector budget [1]. Out of pocket expenditures as a percentage of total health spending was estimated at 32.3% in 2009/10 [2]. With an estimated 14% of the population enrolled in the prepayment schemes, the majority of Tanzanians have to pay for health services at the point of use [1].

In an effort to increase enrolment into prepayment schemes, Tanzania adopted a health financing strategy aimed at achieving universal coverage through the establishment of a number of health insurance schemes targeting specific population groups. The main health insurance schemes currently in operation include the National Health Insurance Fund (NHIF) for public sector employees (and more recently self-employed, employees from private companies, associations and non-governmental organisations, students and clergymen), the Community Health Fund (CHF) for the rural and informal population (and its urban equivalent, TIKA) and the Social Health Insurance Benefit (SHIB) for formal private sector employees who are members of the National Social Security Fund (NSSF). In addition there are various private health insurance funds mostly covering those in the formal sector through their employers and micro-insurance schemes mostly covering informal sector workers [3,4].

Currently, the CHF and NHIF are the predominant funds, covering about 6.6% and 7.2% respectively of the country’s population of 44 million [1,5]. Of the two funds, the NHIF is more successful in enrolment and mobilisation of revenue since membership is mandatory and the funding is automatically deducted from member employee salaries. The challenge remains not only to scale up coverage but also to integrate the existing schemes to achieve the broad level of risk pooling demanded by universal coverage. Inherent in the integration of the existing health insurance schemes is the establishment of a redistributive mechanism that would enable financial transfers to counter differential risk distribution and revenue generation abilities across the schemes. This will guarantee universal access to a basic package of health services, which is the essence of universal coverage.

Redistributive policies demand a level of solidarity, which must exist within a society. Solidarity refers to the willingness to act in the interest of others in need [6,7]. Experience from countries that implemented reforms to achieve greater redistribution demonstrates that a high level of solidarity is a necessary ingredient to the achievement of universal coverage [8]. Support for redistributive policies is grounded on the level of solidarity within the country. Beliefs about solidarity provide an indication of the level of solidarity since these reflect the motivations behind acts of solidarity. Understanding what shapes these beliefs provides important information for developing redistributive policies.

Notions of self-interested or other-regarding preferences determine whether an individual will express support for redistributive policies. Theories that dwell on self-interested behaviour assert that individuals are less interested in the wellbeing of others and are driven primarily by self-gain. This should be important since individuals are affected differently by redistributive policies, with some likely to be net *‘recipients’* in the redistribution, while others more likely to be net *‘payers’* [8-13]. In this regard, it is expected that the less wealthy, older, less educated and non-members (non-insured) are more likely to express support for redistributive policies.

The notion of self-interested preferences has been challenged by evidence that individuals are not driven entirely by self-gain, but tend to exhibit other-regarding preferences as well [14-21]. Other-regarding preferences differ in the motivation behind caring about the welfare of others. This motivation may be based on altruism, which emanates from the ability to empathise with others [6,22-27]. From this theoretical insight, we expect being female and living in a household with members reporting illness to positively influence perceptions towards redistributive policies. Individuals may also extend kindness towards others in order to fulfil social and moral obligations. In this way individuals are more likely to express support for redistributive policies if they have expectations that they will also benefit through reciprocity, praise or avoidance of guilt or social reprimand associated with failing to help others [23,24,28,29].

Contextual factors such as confidence in the institutions given the mandate to implement redistributive policies play a significant role in public support [7,21,30]. Ulrich [7] and Arhinful [30] contend that a lack of confidence in the sustainability and the ability of institutions such as insurance schemes to deliver the expected benefits may negatively influence people’s support for redistributive policies. This paper has used the level of trust in CHF and NHIF management as measures of the confidence in the ability of these institutions to meet expectations of the respective members. It is expected that a high level of trust is likely to positively influence perceptions towards redistributive policies.

This paper analyses the perceptions of CHF, NHIF and non-member households towards cross-subsidisation of the poor as an indication of the level of solidarity and acceptance of redistributive policies. Specifically the paper examines (1) the level of support for redistribution to poorer segments of the population, making comparisons across the three household groups. This was measured by the proportion of the households from each group expressing support towards subsidised CHF membership for the poor and willingness to contribute towards achieving this, (2) the extent of redistribution (the amount of subsidy to the poor) that is likely to be supported and how it compares across the three household groups. This was measured by the proportion of households from each group expressing support for different categories of subsidy, and (3) the determinants of support for redistributive mechanisms. The next section provides the methods used, followed by the results, discussion and finally the conclusion.

## Methods

This study was part of a larger study examining equity in health insurance, conducted in Kongwa and Mpwapwa districts in Tanzania over a period of eight weeks between July and September 2011. The two districts were selected due to their different levels of CHF enrolment, and for convenience in terms of logistics and costs. Kongwa has a total of 63,612 households of which 5,800 (9%) were registered with CHF. Mpwapwa has a total of 78,812 households of which 15,540 (18%) were registered with CHF [31]. The prime economic activity in both districts is agriculture and livestock keeping.

### Sampling method and Sample Size

In each district multistage sampling was used to select first wards, then villages, followed by hamlets^a^ and finally households. A household is defined as a person or group of people related or unrelated who live together and share a common pot of food^b^. For the purpose of this study the household definition was extended to include persons who share the same membership card (CHF households) or are dependents of the same principal member^c^ (NHIF households). The study population comprised all households in the two districts that met this definition. Due to difficulties in identification of households by membership status from the village household register^d^, equal numbers of households were selected from listings of each membership category as follows: CHF households were randomly selected from the CHF register book kept in the health facilities in each ward. This was because health facilities are registration points for CHF membership. The health facilities were selected based on whether the facility catchment area falls within the selected hamlets. To ensure only current CHF members were included, the selection was made from members registered from September 2010 to September 2011.

For NHIF households, a list of all Government institutions in the selected wards or villages was obtained from the District Council, from which all of the NHIF principal members available at the time of the study were selected. This approach was used to ensure achievement of NHIF sample size since there are relatively fewer NHIF members at the ward or village level. Non-member households were randomly selected from the village household register in each of the selected villages. All CHF and NHIF households were omitted from the village register using the list obtained from the facility and District Council respectively before selection of non-member households.

Sample size was calculated based on the reported CHF enrolment rate in each district (11% in Kongwa and 27% in Mpwapwa) and a power of 80%. This resulted in a required sample size of 106 households per group per district. To this, 25 households were added in each group to ensure achievement of sample size. In total 786 households were contacted for interview.

### Data collection

A pre-tested structured questionnaire was administered to the household head or spouse. Three return visits were made to households where members were not available for interview during the first visit. This resulted in a response rate of 88%, with a sample size of 695 households, of which 224 were NHIF members, 233 were CHF members and 238 were non-members. Data were collected on demographic characteristics, employment, education level, family size, the presence of chronic and acute illnesses, membership status, household ownership of assets and consumer durables and perceptions concerning trust in scheme management, subsidised CHF membership for the poor and the willingness to contribute towards this end, and the amount of subsidy that should be considered.

### Study variables

#### Dependent variables

The dependent variables used in the analysis were responses to three statements or questions regarding perceptions towards cross-subsidisation and redistribution to the poor. The responses were measured on the Likert scale [32-34]. The first statement was ‘Poor members of the community should be facilitated to join the CHF without paying the contribution’, for which responses ranged from ‘1 = very strongly disagree to 5 = very strongly agree’. This question measured determinants of the support for subsidised CHF membership for the poor. The second question measured determinants of the amount of subsidy likely to be supported and asked, ‘to what extent should the poor be financially assisted to join the CHF?’ The response ranged from ‘none of the contribution = 1, some of the contribution = 2, half of the contribution = 3, most of the contribution = 4 to all of the contribution = 5’. The third question measured determinants of willingness to contribute towards subsidised CHF membership. For this question, NHIF, CHF and non-member households were each asked two questions: for CHF and non-member households, questions measured willingness to 1) accept and 2) contribute towards subsidised CHF membership for the poor. For NHIF households, questions measured willingness to 1) contribute towards subsidised CHF membership for poor individuals and 2) use of NHIF revenue to cross-subsidise the CHF scheme. For all willingness questions the responses ranged from ‘1 = definitely unwilling to 5 = definitely willing’.

#### Independent variables

The unit of analysis for this study was the household since membership to the CHF is household based. The study used household head characteristics (to represent household decisions) and household characteristics. Household head characteristics variables included age, sex and education level. Age was categorized into three groups (18–35; 36–59; 60 and above). Education level was categorized into four mutually exclusive groups (none, up to Primary, up to Secondary and above Secondary). The household characteristics included three household level variables- wealth status, membership status and presence of household members reporting an illness during the four week (acute) or three month (chronic) recall period used in this study. Wealth status was an asset index used as a proxy for household socio-economic status. Principal Components Analysis was used to develop this index by grouping households into quintiles based on ownership of assets and durable goods. This method has been used in studies done in developing countries to develop indices as proxies for income or wealth status owing to the complexities of determining actual income [35,36]. Membership status was operationalised as ‘1’ for CHF, ‘2’ for NHIF and ‘3’ for non-members. Presence of sick members in the household was operationalised by the means of a dummy variable equalling ‘1’ if an illness had been reported in the household and ‘0’ if there was none. This variable was created from individual household variables that measured the experience of illness among members within each household during the study recall period, which were obtained for addressing other objectives of the larger study. A district variable was also included to control for variations between the two districts.

### Analysis

The study used Kruskal-Wallis (KW) test of equality of populations to compare perceptions among the CHF, NHIF and non-member households. Kruskal-Wallis is a non-parametric version of ANOVA that is used when the dependant variable is ordinal in nature [32,37]. Three generalized ordinal logistic regression models were used to identify factors associated with (1) support for subsiding CHF membership (2) the amount of subsidy supported and (3) willingness to contribute towards subsidised CHF membership for the poor. Ordinal logistic regression is the model of choice when the dependent variable is ordinal in nature, but the Proportional Odds Assumption (or parallel regression assumption) with which it operates often precludes its use [38]. For this reason, the generalized model that relaxes the Proportional Odds Assumption was preferred.

### Ethical considerations

The study proposal was reviewed and approved by Muhimbili University of Health and Social Sciences (MUHAS) Ethics Committee and permission to conduct the study was sought and granted from the relevant authorities at the district, ward and village levels. Informed consent was obtained from all respondents.

## Results

### Descriptive Characteristics of the sample

We obtained complete information on 695 households. Of these households, 224 (32%) were registered with NHIF, 233 (34%) with CHF and 238 (34%) were non-member households. The majority (about 85%) of NHIF households belonged to the highest two wealth quintiles, while 58% of CHF and 56% of non-member households belonged to the lowest two quintiles (ρ < 0.05). CHF households, with a mean household size of 5.3 (Q2 = 5, IQR = 7–4), were larger than NHIF (Q2 = 4, IQR = 6-2) and non- member households (Q2 = 5, IQR = 6–3), which both had a mean size of 4.7 members. Education levels were higher among NHIF heads of households, with 63% having attained secondary or above secondary education, while the majority of CHF (72%) and non-members (64%) household heads had attained primary education. In about 19% of NHIF, 16% of CHF and 25% of non-member households the household heads were female (Table 1).

**Table 1:** **Descriptive Characteristics of households in sample (%)**

	**NHIF (n = 224)**	**CHF (n = 233)**	**Non-member (n = 238)**
**Wealth status**			
Lowest	1.3	29.2	31.5
Second	2.7	29.2	24.4
Third	10.7	23.6	25.2
Fourth	34.4	13.7	12.6
Highest	50.9	4.3	6.3
**Household size (Mean = 4.7 SD = 2.3)**			
1-5	72.3	56.2	69.3
6 and above	27.7	43.8	30.7
**Presence of sick member**			
No	18.3	11.2	20.2
Yes	81.7	88.8	80.8
**Age (head) (Mean = 42.7 SD = 13.5)**			
18-35 yrs	38.8	33.1	30.3
36-59 yrs	56.3	51.5	55.9
60+ yrs	4.9	15.5	13.9
**Sex (head)**			
Male	81.3	84.1	75.2
Female	18.8	15.9	24.8
**Education (head)**			
No education	1.4	20.7	30.7
Up to primary	8.1	72.0	63.8
Up to–secondary	27.9	6.5	5.0
Above secondary	62.6	0.9	0.4

### Household perceptions towards subsidised CHF membership

**Figure 1:**
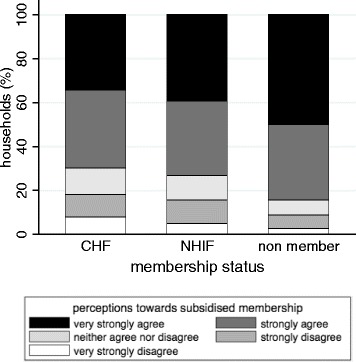
**Household perceptions towards subsidised CHF membership for the poor by membership status (%).**

The median response to the statement ‘Poor members of the community should be facilitated to join the CHF without paying contribution’ was ‘strongly agree’ (Q1 = ‘strongly agree’, Q3 = ‘very strongly agree’). Non-member households expressed the highest support, with a median response of ‘very strongly agree’ (Q1 = ‘strongly agree’, Q3 = ‘very strongly agree’) (Figure 1). KW test showed that non-member households had significantly different responses from CHF and NHIF households (p < 0.05).

Regression analysis showed that compared to CHF members, the odds of NHIF members expressing agreement for subsidised CHF membership for the poor versus disagreement were 2.4 times greater. The odds of expressing agreement versus disagreement were 2.1 times greater for non-members compared to CHF members. Where the household head had a lower level of education, the odds of expressing agreement versus disagreement were 1.5 times higher compared to where the household head was not educated. The odds of expressing agreement versus disagreement were 1.7 times higher when there was a sick member in the household compared to when there was none. The odds of expressing agreement for subsidised CHF membership for the poor versus disagreement were lower for wealthier households in the third (OR 0.4), fourth (OR 0.5) and highest (OR 0. 5) wealth quintiles compared to households in the lowest wealth quintile (Table 2).

**Table 2:** **Generalised ordinal logistic regression model for household perceptions towards subsidised CHF membership of the poor**

	**Odds ratio**	**Robust std. error**
**Membership status**		
CHF^a^		
NHIF	2.414**	0.712
Non-members	2.129***	0.372
**Household wealth**		
Lowest^a^		
Second	0.676	0.167
Third	0.419***	0.104
Fourth	0.533**	0.143
Highest	0.524**	0.148
**Age (household head)**		
18-35^a^		
36-59	0.964	0.157
60+	1.174	0.277
**Sex (household head)**		
Male^a^		
Female	1.159	0.216
**Education level (household head)**		
None^a^		
Up to primary	1.452*	0.319
Up to secondary	1.073	0.371
Above secondary	0.712	0.271
**Presence of sick members**		
No^a^		
Yes	1.736**	0.354
**Trust in fund management**		
Strongly disagree^a^		
Disagree	0.540	0.216
Neither	0.563	0.206
Agree	0.490*	0.179
Strongly agree	1.899	0.863
**District (Kongwa** ^**a**^ **)**		
Mpwapwa	1.201	0.193

*ρ < 0.1, **ρ < 0.05, ***ρ < 0.01; ^a^reference category; response categories dichotomized into the highest category versus the lower categories.

### Household perceptions towards the amount of subsidy

**Figure 2:**
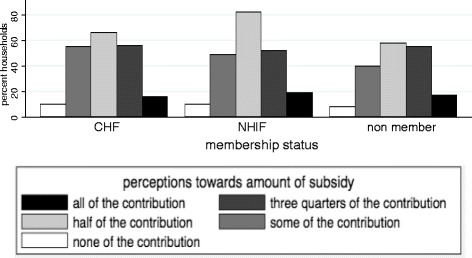
**Perceptions towards the amount of subsidy for CHF membership of the poor by membership status (%).**

The majority of households supported a partial rather than fully subsidised CHF membership for the poor. As illustrated by Figure 2, the median response to the question ‘to what extent should the poor be financially assisted to join the CHF?’ was ‘half of the contribution’ (Q1 = ‘some of the contribution’, Q3 = ‘most of the contribution’). KW test showed differences in responses between the CHF, NHIF and non-member households were not significant.

Regarding the regression results, the odds of expressing support for a higher subsidy for the poor versus a lower subsidy were lower for households in the second (OR 0.8), third (OR 0.5), fourth (OR 0.6) and highest (OR 0.6) wealth quintiles compared to households in the lowest wealth quintile. Where the household head was elderly, the odds of expressing support for a higher subsidy versus a lower subsidy were 1.8 times higher compared to households with younger household heads (Table 3).

**Table 3:** **Generalised ordinal logistic regression model for household perceptions towards the amount of subsidised CHF membership**

	**Odds ratio**	**Robust std. error**
**Membership status**		
CHF^a^		
NHIF	1.187	0.508
Non-members	1.357	0.268
**Household wealth**		
Lowest^a^		
Second	0.838	0.194
Third	0.513**	0.135
Fourth	0.617	0.183
Highest	0.550	0.189
**Age (household head)**		
18-35^a^		
36-59	0.795	0.140
60+	1.775*	0.522
**Sex (household head)**		
Male^a^		
Female	0.790	0.152
**Education level (household head)**		
None^a^		
Up to primary	1.048	0.216
Up to secondary	1.425	0.656
Above secondary	1.425	0.648
**Presence of sick household member**		
No^a^		
Yes	1.196	0.263
**Trust in fund management**		
Strongly disagree^a^		
Disagree	1.159	0.464
Neither	0.795	0.287
Agree	0.905	0.324
Strongly agree	1.548	0.587
**District (Kongwa** ^**a**^ **)**		
Mpwapwa	1.474	0.250

*ρ < 0.1, **ρ < 0.05; ^a^reference category; response categories dichotomized into the highest category versus the lower categories.

### Household willingness to participate in redistributive mechanisms

Table 4 illustrates the households’ willingness to participate in redistributive mechanisms for each membership category. More than 70% of CHF households expressed willingness to continue membership even though poor households would be allowed to join the scheme without paying the contribution. Almost a third of the same households were not willing to increase their contribution to enable poor members of the community to join without paying the contribution.

**Table 4:** **Household willingness to contribute towards subsidised CHF membership for the poor (%)**

**Questions**	**N**	**Not willing**	**Not sure**	**Willing**
**CHF households**				
Are you willing to continue membership if poor members of the community are allowed to join the CHF without paying the contribution?	233	21.7	5.6	73.3
Are you willing to increase your contribution so that poor members of the community are allowed to join the CHF without paying the contribution?	233	44.4	10.7	45.1
**NHIF households**				
Given the choice, are you willing to allow the NHIF scheme to use member contributions to provide additional funds to schemes with poor members?	224	31.7	9.4	59.1
Given the choice, will you be willing to allow part of NHIF funds to be used for paying the contribution on behalf of poorer members of the community in order to enable them to join a health insurance scheme?	224	38.8	11.2	49.1
**Non-member households**				
Are you willing to join the CHF scheme if poor members of the community are allowed to join without paying the contribution?	238	5.5	3.8	90.7
Are you willing to join the CHF scheme and pay higher contribution to enable the poor to join without paying the contribution?	238	6.7	8.8	83.7

Among non-member households 91% expressed willingness to accept subsidised membership for the poor while 84% expressed willingness to contribute towards achieving this. A higher proportion of NHIF households expressed willingness to contribute towards cross-subsidising the CHF scheme as a whole (60%) compared to those that expressed willingness towards subsidising CHF membership for poor individuals (50%).

Results from the regression analysis are presented in Table 5. For CHF households in the third (OR 1.8), fourth (OR 2.3) and highest (OR 9.3) wealth quintiles, the odds of expressing willingness to contribute towards subsidised CHF membership for the poor versus unwillingness were higher compared to those in the lowest wealth quintile. For CHF households where the household heads were aged between 36–59 years, the odds of expressing willingness versus unwillingness were 1.9 timers higher compared to those where household heads were younger (18–35 years). The odds of expressing willingness versus unwillingness were 4.5 times higher for households that expressed confidence in scheme management compared to those that did not. For NHIF households, where household heads had a higher education level, the odds of expressing willingness versus unwillingness were 0.1 times lower compared to those where the household head had a lower education level.

**Table 5:** **Generalised ordinal logistic regression model for households’ willingness to contribute towards subsidised CHF membership**

**Variable**	**CHF**		**NHIF**		**Non-members**	
	**Odds ratio**	**Robust std. err**	**Odds ratio**	**Robust std. err.**	**Odds ratio**	**Robust std. err.**
**Wealth status (lowest** ^**a**^ **)**						
Second	1.455	0.523	0.626	1.073	1.630	0.569
Third	1.806*	0.616	0.800	1.284	0.994	0.368
Fourth	2.335*	1.164	1.904	3.046	1.014	0.434
Highest	9.304***	5.562	2.761	4.459	0.578	0.301
**Age (head, 18–35** ^**a**^ **)**						
36-59	1.877**	0.528	1.108	0.322	0.751	0.237
60+	1.170	0.443	1.078	0.624	1.006	0.450
**Sex (head, male** ^**a**^ **)**						
Female	1.345	0.462	1.617	0.534	0.604	0.200
**Education level (head, no education** ^**a**^ **)**						
Up to primary	0.868	0.234	0.203	0.223	1.179	0.390
Up to secondary	0.797	0.459	0.126*	0.129	0.739	0.571
Above secondary	0.358	0.224	0.120*	0.121	1.050	0.749
**Presence of sick members (no** ^**a**^ **)**						
Yes	0.602	0.243	1.085	0.424	1.437	0.471
**Trust in scheme management (strongly disagree** ^**a**^ **)**						
Disagree	1.473	1.288	1.128	0.698	1.717	1.098
Neither agree nor disagree	1.360	0.979	1.050	0.584	0.688	0.422
Agree	4.455**	3.206	1.327	0.722	1.004	0.606
Strongly agree	2.247	1.873	2.136	2.106	2.465	1.884
**District (Kongwa** ^**a**^ **)**						
Mpwapwa	1.031	0.726	1.525	0.412	0.957	0.258

*ρ < 0.1, **ρ < 0.05, ***ρ < 0.01; ^a^reference category; response categories dichotomized into the highest category versus the lower categories.

## Discussion

This study explored the perceptions of CHF, NHIF and non-member households towards cross-subsidisation of the poor as an indication of the level of solidarity and acceptance of redistributive mechanisms. Our analysis shows that among these households, there is a high level of expressed support for subsidised CHF membership for the poor. However, there is a disparity between expressed support and willingness to contribute towards subsidised CHF membership, especially among CHF and NHIF households. Furthermore the majority of households favour a partial rather than full subsidy for the poor.

### Perceptions towards subsidising CHF membership for the poor

Being a non-member increases the odds of expressing higher support. The higher the education levels of the household head the lower the support for subsidised CHF membership. These findings are consistent with theory that self-interest influences support for redistributive policies. Our findings also corroborate those from empirical studies showing that individuals vulnerable to financial shocks (older, female, low income, uninsured or minimal education) have the highest support for redistributive policies [8-13,39-41]. Guided by self-interest, disadvantaged individuals are likely to benefit more from redistributive policies hence show greater support. Uncertainty about the risk of future loss and the desire for a sense of security makes non-members more likely to support redistributive policies. A higher level of education increases prospects of higher income and better wellbeing, thereby reducing the expected benefits of redistribution. Indeed our results show that the majority (87%) of household heads with secondary education and above are NHIF members, who also happen to be relatively wealthier than CHF and non-member households.

Having a household member reporting illness increases the odds of expressing support for subsidised CHF membership and preference for a higher subsidy. James and Savedoff [13] and Harris et. al [42] also reported similar findings. This finding is also in accordance with the theory of altruistic behaviour which is based on feelings of empathy that enable one to experience what the other is feeling, thus encouraging acts of compassion [22-24,43]. Emphatic attitudes develop as individuals go through life, being exposed to different types of social relationships [6,22,25-27]. Hence, individuals who have interacted with the poor or sick, or who have experienced the responsibility of caring for others, for instance those caring for sick family members, are more likely to empathise with others going through similar experiences [26]. Self-interest may also increase the odds of expressing support, since households with a sick member would benefit more from subsidised membership than those without. However, our findings also show that households reporting a sick member included wealthy households as well as NHIF households that would not be eligible for subsidised CHF membership. Therefore it more likely that support for subsidised CHF membership stemmed from altruism rather than self-interest.

Except for experience of illness in a household, other variables do not influence perceptions towards the amount of subsidy that should be given to the poor. Despite this, our findings showed that the majority expressed support for subsidising a partial, rather than the full membership contribution. This corroborates findings of James and Savedoff [13] and implies that there are sentiments that the poor should make some contribution towards securing their health. It has been recognised that people tend to oppose redistributive policies when they feel that it reduces incentives for the poor to bear their share of the burden [25]. It could be that people want to avoid the ‘free rider effect’ of providing the full subsidy, where the beneficiaries of subsidised membership may take advantage of the policy. This could give the impression that not all poor people are too destitute to pay even a portion of the contribution. It could also be that the poor are expected to make a contribution to demonstrate the ‘value’ they place on the subsidised membership received. However, the findings depart from the experience of Ghana and Rwanda where fully subsidised membership for the poor has been implemented [44,45]. This raises questions about why the sentiments towards a full subsidy would differ between countries of similar developing country context. A better understanding of the underlying reasons for supporting a partial rather than full subsidy is required.

These findings imply that while the majority of households generally support subsidised CHF membership, the degree of support differs depending on the perceived benefits and the amount of subsidy under consideration. Individuals who may not directly benefit from such a policy (the insured, higher educated) are likely to express less support for subsidised membership. It has been argued that other-regarding behaviour is motivated by expectations of reciprocated kindness, of praise and recognition, or as a way to avoid feelings of guilt or social isolation associated with failing to help others [23,24,28,29]. Hence, other-regarding preferences are often viewed as indirect expressions of self-interest [30,39]. From this perspective, support for redistributive policies can be promoted by focusing on extended family relations that are often the norm in African societies, and where the better off are obliged to financially assist their poor relatives to access health care. Self-interested individuals can be persuaded to support redistributive mechanisms by emphasising the indirect benefits. Also, the findings suggest that a fully subsidised membership is likely to be met with resistance by the majority. Careful consideration should be given to the amount of subsidy, such that there is minimal if any resistance but at the same time the poor are enabled to join the CHF. Given that an estimated 34% of the population [46] will need fully subsidised membership if Tanzania is to achieve universal health coverage, this could prove quite a challenge. This underscores the need for a deeper understanding of the perceptions towards the amount of subsidy.

### Willingness to accept and contribute towards subsidised CHF membership for the poor

Willingness to accept poor members joining the CHF without paying the contribution demonstrates some degree of positivity towards solidarity. This was higher among non-member households, which should not be surprising given that many of them, being in the poorest two quintiles, could see themselves as net *‘recipients’* of redistributive policies. However, what is surprising is that these are the same households that decided not to join the scheme in the first place. The findings show that more than 80% of non-member households agreed to join if poorer groups receive a subsidy, while less than 40% were from households in the lowest wealth quintile. Perhaps misinterpretation of the question may explain this anomaly. It could be that most respondents interpreted the question as asking for their willingness to join the scheme with subsidised membership rather than their willingness to join (without subsidy) while poor households receive a subsidy. Whatever the case, the question of high willingness to join the CHF at the mention of a subsidy still remains; especially for wealthy households where we can assume affordability is not an important barrier to joining the scheme. There may be trust issues regarding quality of care and scheme management, such that they are only willing to join with a subsidised membership contribution. Could the disinclination to join the CHF be a demonstration of their lack of solidarity with the rest of the community? This could be the case if these households have a lower propensity to need health services and/or can afford to pay for better quality of care at private facilities that do not provide services to CHF members. A deeper understanding of the motives behind these responses is necessary but beyond the scope of this paper.

It is also interesting to note that among CHF and non-member households, willingness to contribute to subsidised membership for the poor is lower than willingness to accept it. This implies that although the majority support cross-subsidisation of the poor they may not think it is their responsibility to contribute towards their welfare. A study in Ghana found that people assumed it is the responsibility of the government, charity organisations, or the church rather that the community to provide financial assistance to the poor when they need health care [30]. The same may be the case for our respondents, but further research is needed to understand their views on the role of government or other institutions versus the community in assisting the poor.

NHIF households, who may see themselves as the net ‘*payers’* in the redistribution, thus, sacrificing the most, show moderate willingness to contribute towards a redistributive mechanism. Indeed for the NHIF, compromises may include an increased salary deduction with no direct benefits, while for CHF members and non-members, this may entail paying higher contributions but with additional funding for the scheme. These findings concur with theoretical arguments that preferences for redistribution are motivated by self-interest. Our results also agree with those of Goudge et. al [41] who pointed out that high income groups may be guarded about the financial burden they would have to bear if they support redistributive policies.

The regression analysis demonstrates that among CHF households, being wealthy and young is associated with willingness to contribute to redistributive mechanisms. This finding is in keeping with the Becker’s [47] theory, which states that individuals are altruistic, but respond to the cost of giving. This implies that ability to extend help to those in need affects their willingness to contribute to redistributive mechanisms. Therefore households in the lowest quintile may have the highest support for subsidised membership of the poor but it may not be within their ability to contribute and so most refrain from making a commitment to that end. The same may apply for households headed by older people who are either retired or not earning a living and may not be in a position to help others, much as the desire exists. Although the results are not significant, the same trends are observed for NHIF households. Also agreeing with Becker [47], the analysis predicts among non-member households, a lower willingness of female-headed households to contribute towards subsidised CHF membership for the poor. Indeed, a higher proportion of female-headed households in the lowest wealth quintile and with lower education levels were non-members, which was not the case for CHF and NHIF households. In a similar study, Goudge et. al, [41] also found that men were more willing to pay for health insurance for the poor.

Trust in scheme management is associated with increased willingness among CHF households, although there does not appear to be a consistent gradient. Indeed trust in scheme management has been cited as an important factor influencing acceptance of redistribution [7]. This is because individuals may support such policies with the expectation of benefitting at some point in the future, for instance when retired or when older and more at risk of needing expensive treatment. Therefore, individuals must have confidence that institutions will still be able to deliver the expected benefits at that point in the future. Trust in insurance schemes goes beyond the scheme management to include the quality of health services [48]. Past experience of health care services and management of the scheme is likely to influence their willingness to contribute to redistributive mechanisms. Furthermore, the level of trust in the CHF will influence whether or not individuals feel that their contributions will indeed be put to intended use. Individuals who perceive government institutions to have limited or non-existent accountability will not have confidence that the poor will benefit from their contributions. Trust also influences enrolment into the CHF, hence the constraints of enforcing mandatory health insurance coverage in the Tanzanian context and the necessity of high enrolment for greater redistributive potential, emphasizes the importance of building trust in scheme management. It is therefore important that efforts for improvement be directed at quality of health services, as well as transparency and accountability in management of the scheme.

This study sought to explore whether or not a redistributive policy between the CHF and NHIF will be supported. Since this has not been experienced the survey questions were designed to assess beliefs about notions of solidarity with the poor and whether or not individuals are willing to contribute to achieve it. Therefore, we cannot confidently say that the perceptions expressed will translate into actual practice of solidarity. Rather, our findings serve as an indication of solidarity, based on expressed feelings of solidarity. Moreover, the quantitative approach used meant that the study did not capture the diverse beliefs about solidarity, for instance the motive behind support for a partial rather than full subsidy, willingness to accept and contribute towards its achievement, the specific amount people would be willing to contribute, and the responsibility for assisting the poor to access health care. Triangulating the study findings with those from qualitative methods would have provided a deeper understanding of the beliefs of solidarity. In addition, the study did not have a clear definition of ‘poor members of the community’, but was based on the assumption that the poor were those who were ‘financially poor’ and could not afford the membership contribution. This may have led to the respondents having different interpretations of who is poor, which may have affected the results. However, since the study focus was on enabling those who could not afford to pay the CHF contribution, it is likely that the majority of the respondents had the same interpretation of who is poor.

## Conclusions

This study has shown that the majority expressed support for redistributive policies, but the findings suggest that the support and willingness to contribute to its achievement are influenced by the perceived benefits, amount of subsidy considered, and trust in scheme management. These present three important issues for consideration when designing redistributive policies. First, perceived benefits of redistributive policies are an important consideration given the key position of those who may not benefit directly, that is, NHIF and CHF members and wealthy non-member households. Second, while a partial subsidy is more likely to be supported, the amount should not be too low such that the poor cannot meet their share of the contribution. Lastly, given the interdependence of quality of health services and scheme management, efforts are needed to enhance confidence in the scheme to provide needed services and manage redistributed resources for the benefit of the poor.

Development of redistributive policies will benefit from information from qualitative studies focusing on views on the motive behind support for a partial subsidy, willingness to accept and contribute towards its achievement, the specific amount people would be willing to contribute and the responsibility for assisting the poor to access health care and the relative importance of self-interest or altruism in explaining support for and willingness to participate in redistributive policies.

## Endnotes

^a^In Tanzania, districts are organised into divisions, which in turn are divided into wards. Within each ward, there are a number of villages, which are also divided into hamlets. Depending on the ward and health infrastructure, one health facility may have a catchment area of one or more villages.

^b^This definition is based on that used by the 2010 Tanzania Demographic and Health Survey (TDHS).

^c^Principal member is the contributing member of the NHIF, usually the head of household or spouse.

^d^Each village has a list of all households registered at the office of the Village Executive Officer. This list is broken down by hamlet but does not show membership status.
